# Synthesizing evidence from clinical trials with dynamic interactive argument trees

**DOI:** 10.1186/s13326-022-00270-8

**Published:** 2022-06-03

**Authors:** Olivia Sanchez-Graillet, Christian Witte, Frank Grimm, Steffen Grautoff, Basil Ell, Philipp Cimiano

**Affiliations:** 1 Semantic Computing Group, Cluster of Excellence Cognitive Interaction Technology (CITEC), Bielefeld University, Bielefeld, 33619 Germany; 2 Zentrale Notaufnahme, Klinikum Herford, Herford, Germany; 3 SIRIUS labs, Oslo University, Oslo, Norway

**Keywords:** Argument-based systems, Aggregation of clinical trial evidence, Evidence synthesis, Systematic review automation

## Abstract

**Background:**

Evidence-based medicine propagates that medical/clinical decisions are made by taking into account high-quality evidence, most notably in the form of randomized clinical trials. Evidence-based decision-making requires aggregating the evidence available in multiple trials to reach –by means of systematic reviews– a conclusive recommendation on which treatment is best suited for a given patient population. However, it is challenging to produce systematic reviews to keep up with the ever-growing number of published clinical trials. Therefore, new computational approaches are necessary to support the creation of systematic reviews that include the most up-to-date evidence.We propose a method to synthesize the evidence available in clinical trials in an ad-hoc and on-demand manner by automatically arranging such evidence in the form of a *hierarchical argument* that recommends a therapy as being superior to some other therapy along a number of key dimensions corresponding to the clinical endpoints of interest. The method has also been implemented as a web tool that allows users to explore the effects of excluding different points of evidence, and indicating relative preferences on the endpoints.

**Results:**

Through two use cases, our method was shown to be able to generate conclusions similar to the ones of published systematic reviews. To evaluate our method implemented as a web tool, we carried out a survey and usability analysis with medical professionals. The results show that the tool was perceived as being valuable, acknowledging its potential to inform clinical decision-making and to complement the information from existing medical guidelines.

**Conclusions:**

The method presented is a simple but yet effective argumentation-based method that contributes to support the synthesis of clinical trial evidence. A current limitation of the method is that it relies on a manually populated knowledge base. This problem could be alleviated by deploying natural language processing methods to extract the relevant information from publications.

## Background

The evidence-based medicine (EBM) paradigm fosters the use of the best available evidence when making decisions in treating individual patients [1]. Best evidence mainly refers to the evidence in the form of randomized clinical trials (RCTs) (Cf. GRADE guidelines [2]). Identifying such best evidence requires the aggregation of the information from multiple clinical trials, an activity that is typically performed in the form of systematic reviews and/or meta-analyses [3]. Yet, the process of extracting and aggregating evidence from multiple published trials represents a significant effort.

In order to help reduce the effort in aggregating evidence as a crucial step in elaborating a systematic review, in this paper we present an argument-based approach that automatically generates a conclusion from a given body of semantically captured clinical trials. The main goal of a systematic review is to identify which of a number of existing treatments is superior to other treatments. Therefore, our methodology automatically generates a conclusion together with a justification in how far one treatment can be seen as superior to another one, where the backing evidence comes from a knowledge base in which clinical trials are semantically described. The conclusion and corresponding justification are provided in the form of a tree consisting of an overall conclusion on the superiority of one treatment over another at the root of the tree, and interim conclusions regarding the hierarchically ordered sub-dimensions along which the treatments can be compared. The sub-dimensions are ordered in a *dimension tree* in which the nodes correspond to standard primary and secondary endpoints considered in a given therapy area (e.g., safety → hypoglycemia → nocturnal hypoglycemia). Our proposed methodology generates such a hierarchical, tree-shaped argument from a given knowledge base automatically, but most importantly makes the process of generating such a conclusion interactive and dynamic. By changing the weights, and thus the relative importance of each comparative dimension (e.g., weighting safety higher than efficacy), a user can perform a **sensitivity analysis** to understand under which conditions and assumptions a certain treatment can be assumed to be superior to another one. The method is also dynamic in the sense that it can incorporate new evidence as it becomes available instantly, and the users can inspect how the newly added evidence affects the overall conclusion. We thus call our method *Dynamic Interactive Argumentation Trees* (DIAeT).

Little work exists on addressing reasoning through argumentation on the analysis and synthesis of clinical trial information. There have been efforts on using argumentation theory in the biomedical domain that rather focus on decision-making and the explanation of individual treatments (e.g., [4, 5]), but not on the synthesis of information of various clinical trials. The argumentation approach of Hunter and Williams [6] is the closest to the goal of our method of supporting the synthesis of clinical trial evidence and generating conclusions in different scenarios considering the expert users’ preferences on the studied clinical trial endpoints. Hunter and Williams’ approach is a formal framework that uses an abstract argumentation model to generate and aggregate arguments for claiming superiority of treatments based on the provided evidence, and considering preferences over the outcome indicators. The framework consists of a directed graph where the nodes are arguments and the arrows are attack relations. The groups of non-conflicting arguments are formed by accepting/rejecting arguments according to a defined semantics of acceptance. The evidence consists of relative risk values for clinical outcome indicators (or endpoints) with respect to the outcomes and side effects of the applied drug treatments (or control) for a given health problem.

In contrast to Hunter and Williams’ approach, our method does not rely on abstract argumentation but rather on Toulmin-style argumentation [7], which is a practical approach to argumentation by focusing on the justificatory aspects, such that no explicit distinction between attacking and supporting arguments is needed.

Other methods to synthesize clinical trial evidence that do not follow an argumentation-based approach use statistical approaches. For example, the Aggregation Data Drug System (ADDIS) [8] supports the generation of network meta-analyses through statistical methods (e.g., Bayesian meta-analysis) and the quantitative benefit-risk analysis of treatments (e.g., using stochastic simulation). ADDIS relies on a data model in XML format that does not fully model clinical trials but supports evidence synthesis. It also counts on a semi-automatic procedure to import clinical trial information from existing data sources into their XML model. Our approach differs from ADDIS in that it does not apply statistical methods to determine the superiority of treatments. Instead, it uses argument concepts to infer conclusions on the superiority of therapies that can be further analyzed by expert users. Besides, our method uses a knowledge base that semantically represents the clinical trials and that allows querying such information more richly than in XML data sources.

While ADDIS and Hunter and Williams’ framework are formally rigorous, we believe that the conceptual simplicity of our method is a useful feature as it is easy to adapt to other types of interventions and health conditions and it is straightforward to specify preferences between dimensions via weights on the single dimensions.

In this paper, we describe the method sketched above for the automatic generation of conclusions that summarize the evidence available in a set of clinical trials. We describe the method technically, in particular showing that it supports sensitivity analysis. As a proof-of-concept, we demonstrate that the main conclusions of two published systematic reviews can be reproduced with our approach. We further present the results of a usability study showing that medical practitioners find the tool easy to use, and they understand how conclusions are generated from the available evidence.

## Method

Our method generates a conclusion from the existing evidence with respect to the superiority of a given treatment in comparison to another treatment. While we focus on the direct comparison between two treatments in our exposition of the method, the approach can be extended to comparing multiple treatments.

The conclusion generated has the form of a tree in the sense that it consists of an overall conclusion about the superiority of the treatment at the root level which points to several children representing the (interim) conclusions for specific comparison criteria. Take the following example of an automatically generated conclusion comparing two types of insulin, the Neutral Protamine Hagedorn insulin (NPH) and insulin glargine (IGlar) as treatments for Type 2 Diabetes Mellitus (T2DM):

↦ IGlar is overall superior to NPH insulin in terms of safety (considering nocturnal hypoglycemia) and efficacy (considering HbA1c reduction) when weighted equally.
⇒ IGlar is superior to NPH insulin in terms of efficacy.
  → Benedetti et al. [9] show that IGlar is superior to NPH insulin in reducing HbA1c.
  → Hsia et al. [10] show that IGlar is NOT superior to NPH insulin in reducing HbA1c.
  → “*n* other arguments from corresponding studies” show that IGlar is superior to NPH insulin in reducing HbA1c.
⇒ IGlar is superior to NPH insulin in terms of safety.
  → Benedetti et al. [9] shows that IGlar is superior to NPH insulin in terms of nocturnal hypoglycemia.
  → No study shows that IGlar is NOT superior to NPH insulin in terms of nocturnal hypoglycemia.
  → “*n* other arguments from corresponding studies” show that IGlar is superior to NPH insulin in terms of nocturnal hypoglycemia.

The overall conclusion (pointed with ↦) claims the superiority of IGlar with respect to NPH insulin when efficiency and safety are weighted equally. As a justification of this overall conclusion, we have the (interim) conclusions/arguments claiming superiority of IGlar with respect to NPH insulin in terms of safety and efficacy, respectively (pointed with ⇒). As a child of the (interim) conclusion claiming the superiority of IGlar compared to NPH insulin with respect to efficacy, we have an argument claiming superiority of IGlar compared to NPH insulin in terms of higher effectiveness in reducing HbA1c. As a child of the (interim) conclusion regarding the superiority of IGlar compared to NPH insulin regarding safety, we have an (interim) conclusion that IGlar is superior to NPH insulin regarding the reduction of nocturnal hypoglycemia. Finally, the children of the last two (interim) conclusions point to claims in specific publications backing up the claim of superiority with respect to higher effectiveness in reducing HbA1c as well as reducing cases of nocturnal hypoglycemia. Each node in the argumentation tree thus represents an (intermediate) conclusion that is justified by the nodes below, until reaching the claims of specific publications. The specific conclusions derived from claims of specific publications are called *Atomic Arguments* while the arguments generated by our method and aggregating the results across clinical trials are called *Aggregated Arguments*.

The method relies on a knowledge base in which all relevant trials have been semantically described in the Resource Description Framework (RDF) following the C-TrO Ontology [11]. We note that any other correspondingly expressive ontology could be used. The argumentation tree is computed using a recursive procedure starting from the root of the tree, invoking procedures to generate the children arguments recursively. Thus, the first arguments/conclusions that are generated are the atomic arguments, with information flowing up to higher levels of the tree where the information is aggregated.

In the following, we first describe the C-TrO ontology and how it is used in our approach to semantically capture the results from clinical trials in a knowledge base. We further describe the procedure for automatically generating the Dynamic Interactive Argumentation Tree (DIAeT) representing the hierarchical conclusion on the basis of the given knowledge base. We present the relevant definitions and other important concepts needed to expose our approach before describing the method formally. We also hint at requirements that NLP methods that automatically extract evidence from publications need to fulfill.

### The C-TrO ontology and knowledge base

**Fig. 1:**
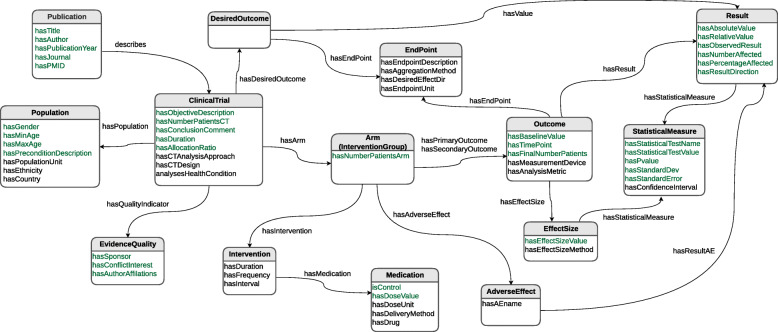
Diagram of the main classes of C-TrO. Data properties are in green and Object properties in black. The arrows start at the domain classes and end at the range classes

In order to provide a proof-of-concept for our method, we have manually populated an RDF knowledge base following the structure of the C-TrO ontology [11]. Existing clinical ontologies [12–15] have been designed to support the searching, question formulation, and retrieval of evidence from the scientific literature, and focus on a coarse-grained representation of the PICO elements. For example, in the PICO ontology [14], the outcomes are represented as textual descriptions but not in more detail as numerical values for each result of the interventions. Although the Study Cohort Ontology (SCO) [15] considers some pertinent entities for clinical trials such as diseases, drugs, and populations, it does not include all the entities and relationships useful for clinical trial synthesis (e.g., quantitative results of endpoints). In contrast, C-TrO was designed to support the aggregation/synthesis of clinical evidence. It describes fine-grained information about results comparing a certain interventional group (or arm) to a baseline condition and allowing to claim differences from the mean, reductions, size-of-effect, etc. Figure 1 shows the schema of C-TrO used in this work.

**Fig. 2:**
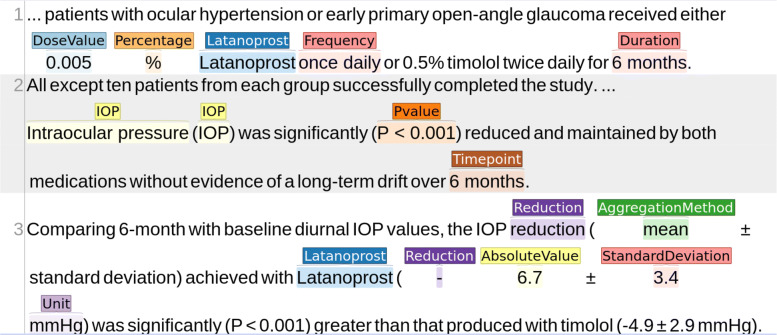
Annotated excerpt from a glaucoma clinical trial. Only the pieces of information related to the *latanoprost* intervention and one of its outcomes are annotated for illustrative purposes. (The annotations were made with INCEpTION [31])

C-TrO has been developed as a general schema to represent the design and results of clinical studies, and it is independent of a particular data source. We used Protégé [16] to populate the C-TrO knowledge base by manually extracting the information from the clinical trials studied in the meta-analyses on glaucoma and on T2DM that are included in the use cases presented later. As a result, the information of the relevant clinical trials is captured in the form of RDF triples in the knowledge base. The example in Fig. 2 illustrates part of the description of the results in a published clinical trial on glaucoma [17] (PMID 8628544) that has been formalized in the knowledge base. An excerpt of the triple representation describing the corresponding study in RDF is given in Table 1. The full RDF file can be downloaded from the repository indicated in “Availability of Data and Materials”. Once the information is in the knowledge base, the method, implemented as a tool, retrieves the information with a SPARQL query formed according to the parameters selected in the user interface (see Table 2). The retrieved information is the base evidence used in the construction of the DIAeTs.

**Table 1:**
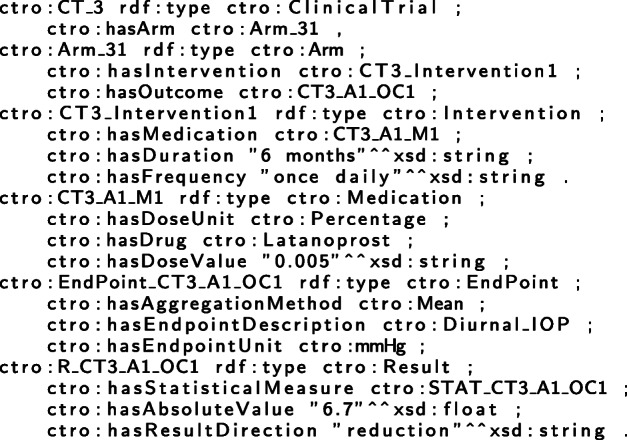
Triples corresponding to some information from the clinical trial PMID 8628544

**Table 2:**
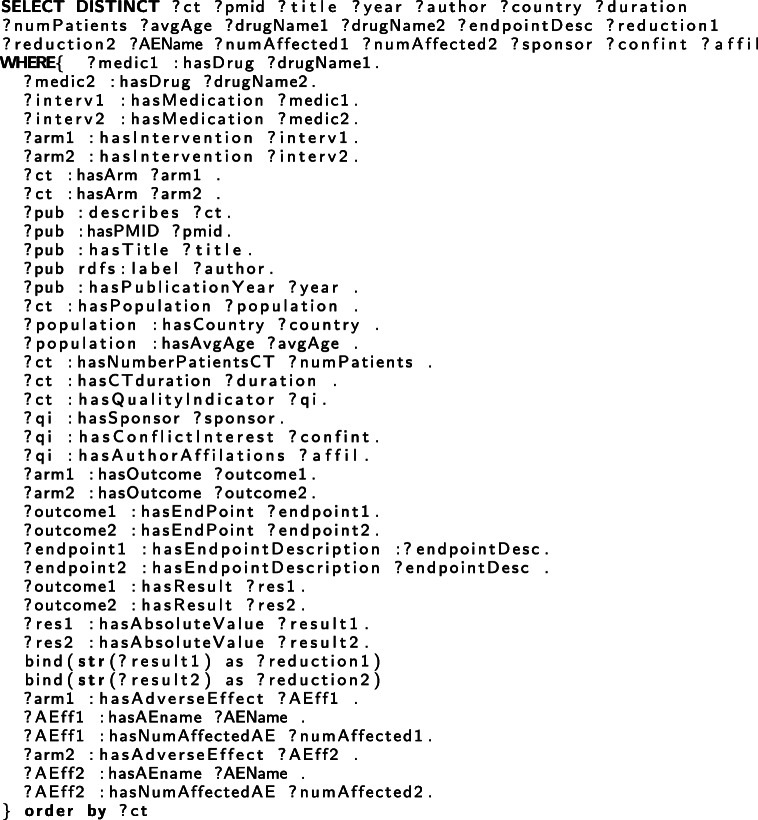
SPARQL query to retrieve clinical evidence from the C-TrO knowledge base. The values for variables ?*drugName*1,?*drugName*2,?*endpointDesc*, and ?*AEName* are passed from the system

### Natural language processing (NLP) requirements

While we have modeled the evidence manually for this work, the option of applying NLP methods to extract the evidence from publications automatically is appealing. However, there are a number of requirements to be fulfilled by such NLP methods to be applicable in our context. Such methods should be able to generate a machine-readable representation of a publication that comprises the study design, population characteristics, in particular the condition, inclusion and exclusion criteria, age of participants, duration of a study, and most importantly the arms of the study with the corresponding treatment information including dosage information, frequency of application, etc. Further, the central outcomes including values and units need to be extracted for every endpoint, primary and secondary, comparing the different arms. Corresponding semantic medical vocabularies such as the Medical Subject Headings (MeSH) or the International Classification of Diseases (ICD) should be used to normalize treatments, conditions, etc.

### Definition of concepts

**Arguments** Structured arguments consist of a set of premises and a conclusion or claim in which the premises are statements that support the conclusion. In our approach, **arguments** represent a valid conclusion about the superiority of a therapy/intervention that can be reached on the basis of the clinical trial evidence available in a given knowledge base. The arguments can be nested in the sense that each argument consists of a set of premises and a conclusion where each premise itself can be an argument. In this context, we define an *argument* as a 5-tuple (*C*,*t*,{*t*_1_,...,*t*_*n*_},*d*,{*p*_1_,...,*p*_*m*_}) where:

- *C* is a conclusion about the superiority of therapy *t* compared to other therapies {*t*_1_,...,*t*_*n*_},
- *d* is a dimension (i.e., a clinical endpoint) along which therapy *t* is compared to the alternative therapies,
- {*p*_1_,...,*p*_*m*_} is a set of premises from which the conclusion follows. A premise *p*_*i*_ can be an argument or a set of facts from a knowledge base.

For demonstrative purposes, in the remainder of this article we only consider a singleton set for the competing therapies, i.e., {*t*^′^}. We distinguish between two types of arguments: **Atomic Arguments** (*AtA*s) and **Aggregated Arguments** (*AgA*s).

**Fig. 3:**
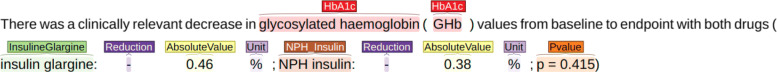
Example of an annotated statement that involves an atomic argument. “%" refers to the Diabetes Control and Complications Trial (DCCT) unit used to measure HbA1c levels. (Annotations made with INCEpTION [31])

**Atomic Arguments (*AtA*)** represent a single result from a published clinical trial that warrants a superiority conclusion with respect to a specific dimension *d*. An example of an atomic argument is in the annotated statement taken from a published clinical trial (PMID 12734781 [9]) depicted in Fig. 3. This statement claims that insulin glargine (IGlar) is superior in reducing HbA1c to NPH insulin, since it decreases the HbA1c levels in a significant amount from the baseline (i.e., 0.46 vs 0.38, where “-” refers to reduction). In this example, the comparative dimension *d* is *HbA1c reduction*.

**Aggregated Arguments (*AgA*)** are arguments whose premises are atomic arguments or other aggregated arguments, and their conclusion is an aggregated claim. An example of an aggregated argument would be an argument generated by considering the results from multiple papers comparing the IGlar therapy to the NPH insulin therapy, claiming that in a certain percentage (e.g., 80%) of studies, it has been demonstrated that IGlar *is superior* to NPH insulin in terms of *HbA1c reduction*.

**The dimension tree** is a tree that hierarchically encodes the relevant dimensions to be used to compare to treatments in a tree representation. In the dimension tree, each node corresponds to a certain **dimension** (i.e., clinical endpoint) that can be used to compare therapies with each other. The dimensions are hierarchically ordered along the tree in the sense that there is a specialization/generalization relation between children and parent nodes. For example, the dimension *safety* for a given treatment, could have the sub-dimensions “risk of mortality”, “mild/high pressure”, and “nausea”. Each dimension is associated with a **weight** according to the importance given to the corresponding clinical endpoint.

**Fig. 4:**
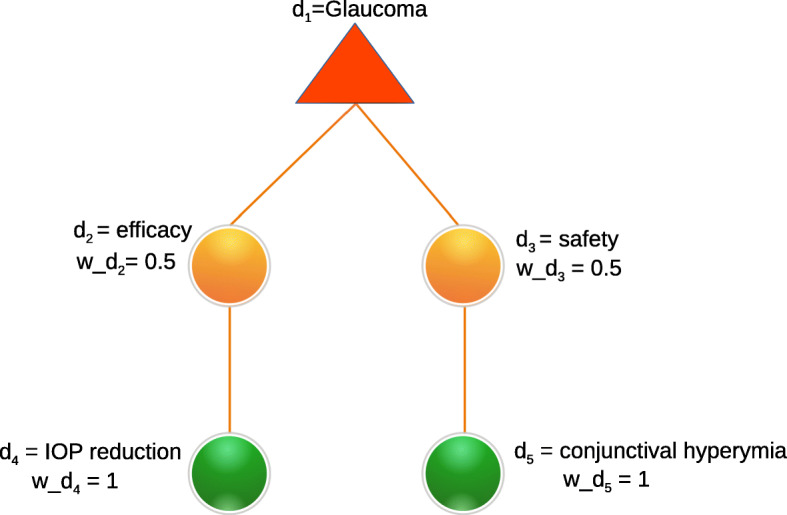
Example of a dimension tree for glaucoma. The tree contains the dimensions: efficacy, safety, IOP reduction and conjuntival hyperaemia, and their respective weights

The dimension tree is specific to a certain therapeutic area or indication, representing the community consensus on which endpoints are relevant and accepted as evidence in clinical trials. An example of a dimension tree is depicted in Fig. 4.

**Degree of confidence** Since the clinical trial evidence may be affected by inconsistencies or contradictions (i.e., called ‘attacks’ in the computational argumentation literature [18]) by other pieces of evidence, the conclusion about the superiority of one therapy over other therapies may not be unanimously warranted. To address this, we indicate the *degree of confidence* to which the conclusion of an argument is warranted by the premises. This is the certainty/confidence that a certain claim holds by quantifying the number of studies in which the given results have been shown in relation to the overall number of studies.

Being $\llbracket \mathcal {A}\rrbracket $ the degree of confidence of an argument $\mathcal {A}$, we compute the degree of confidence for a specific claim as follows:

*For atomic arguments*, the degree of confidence *⟦AtA⟧* is 1 if a certain study claims superiority of *t* compared to *t*^′^, and 0 otherwise. That is, 1 denotes a supporting statement, and 0 a contradictory one. For example, when comparing IGlar to NPH insulin, for the atomic argument *AtA*_1_ “Benedetti et al. [9] show that IGlar is superior to NPH insulin in reducing HbA1c”, *⟦AtA*_1_*⟧*=1, while for the atomic argument *AtA*_2_ “Hsia et al. [10] show that IGlar is NOT superior to NPH insulin in reducing HbA1c”, *⟦AtA*_2_*⟧*=0.

*For aggregation arguments*, the degree of confidence written as *⟦AgA⟧* is computed as follows:

1 $$ \llbracket AgA \rrbracket = \frac{1}{Z} \sum_{A_{i} \in \{A_{1},\dots,A_{k} \}} w_{A_{i}} * \llbracket A_{i} \rrbracket  $$

Where {*A*_1_,…,*A*_*k*_} is the set of arguments to be aggregated, $\phantom {\dot {i}\!}w_{A_{i}}$ is the weight of the corresponding dimension (assigned in the dimension tree) for the argument *A*_*i*_ being aggregated, and the normalization factor *Z* is:

2 $$  Z = \sum_{A_{i} \in \{A_{1},\dots,A_{k} \}} w_{A_{i}}  $$

Note that the weights are non-negative values and *⟦AgA⟧*∈[1,0] since the weights are normalized.

**Confidence acceptance threshold** As in the general case the evidence can not be assumed to be homogeneous with studies having contradictory findings, our method introduces a confidence threshold *τ* that needs to be reached or surpassed by the confidence of an aggregation argument to be accepted. The interpretation of the threshold corresponds to the relative share of clinical studies that need to agree on a certain result (e.g. superiority of therapy A compared to B for a specific outcome).

If a user wants to consider only results for which no contradictory evidence exists, then the threshold has to be set to 1. In the general case, a user can set the threshold to a value corresponding to the inconsistency he/she is willing to accept regarding the conclusion. The default value for the threshold is 0.5 (or 50%), indicating that at least half of clinical trials need to agree on a certain outcome. A user can set the threshold higher to impose a stricter requirement on the homogeneity of the evidence.

### Construction of a DIAeT

The DIAeT is a tree where the nodes represent arguments and the edges connect arguments with sub-arguments. The atomic arguments correspond to the leaf nodes and the aggregated arguments to the inner nodes. The children of a node are sub-arguments (or sub-conclusions) that occur in the premises of the given argument node.

The construction of the DIAeT is driven by a given dimension tree and follows a recursive procedure. Each node recursively calls the procedure that generates sub-arguments that support the conclusion at the node in question. The procedure starts at the general conclusion located at the root node of the argument tree and stops at the leaf nodes that correspond to atomic arguments.

The end of the recursion coincides with the generation of as many atomic arguments =(,*⊔*,*⊔*^′^,*⌈*,*{⊣⌋⊔∫*_*⌈*_(*√*,*⊔*,*⊔*^′^)) for a **leaf dimension node***d* for each publication *p* that compares treatments *t* and *t*^′^ with respect to dimension *d*, where *facts*_*d*_(*p*,*t*,*t*^′^) represents the evidential facts in publication *p* that justifies the claim of superiority of *t* over *t*^′^ w.r.t. *d*.

The depth of the generated argument tree is bound by the depth of the dimension tree, which is a finite tree. Therefore, the recursive process can never fall into an endless loop and stops at the leaf nodes of the dimension tree.

The instantiation of atomic arguments follows the **superiority criteria** defined for each dimension. These criteria state how superiority is considered based on the evidence retrieved from the knowledge base. For example, a superiority criterion for the dimension *efficacy* in the case of T2DM would be to consider as superior the drug treatment that reduces the highest amount of protein HbA1c.

At an inner node *d*_*inner*_ of the dimension tree with children *d*_1_,...,*d*_*n*_, an aggregated argument is constructed as follows:

$\phantom {\dot {i}\!}AgA_{inner}=(C_{inner},t,\{t'\},d_{inner},\{A_{d_{1}},...,A_{d_{n}}\})$, where $\phantom {\dot {i}\!}A_{d_{i}}$ are atomic arguments (if *d*_*i*_ is a leaf dimension) or aggregated arguments (else). In both cases $\phantom {\dot {i}\!}A_{d_{i}}$ claims superiority of treatment *t* over treatment *t*^′^ with respect to dimension *d*_*i*_.

An aggregated argument is **accepted** if its degree of confidence *⟦AgA⟧* is not less than the user-defined (or default) acceptance threshold *τ*. Thus, if the degree of confidence *⟦AgA⟧*≥*τ* for *t*, then the **conclusion** (*C*) will state that treatment *t* is superior to treatment *t*^′^ w.r.t. dimension *d*_*i*_. Afterwards, the generated arguments are verbalized by domain-specific templates. The procedure to construct a DIAeT is summarized in Algorithm 1.


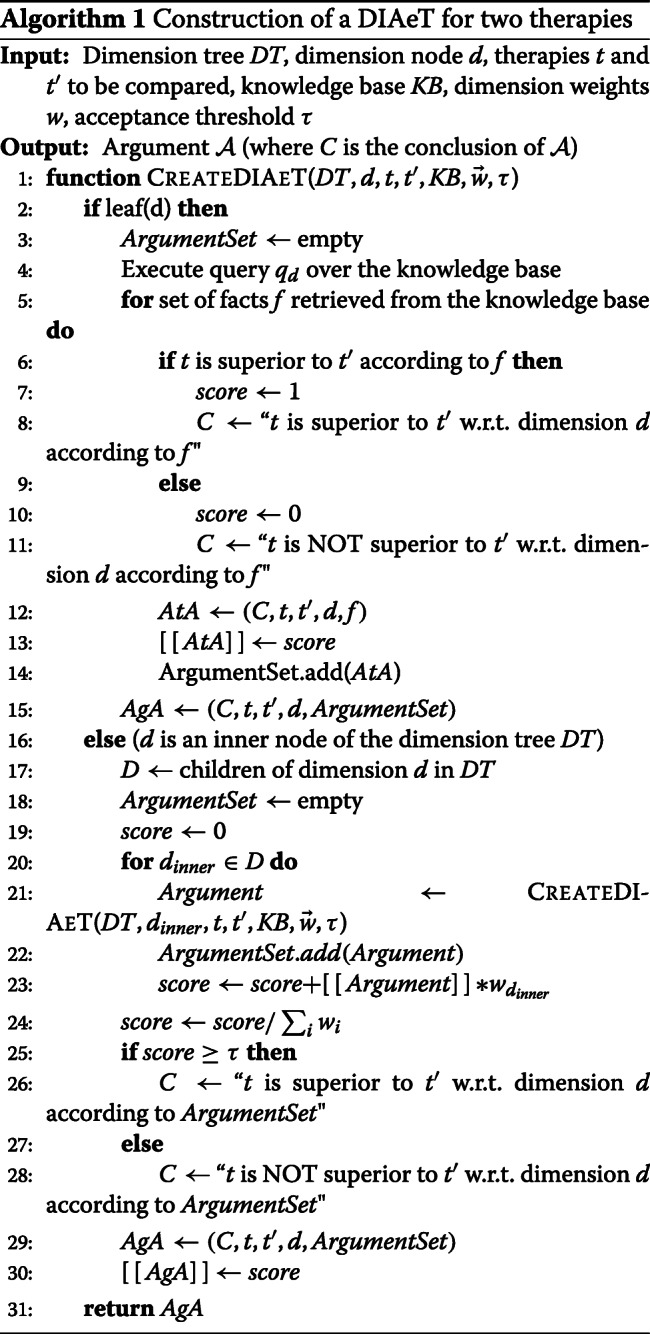


### Example of the construction of a DIAeT

Figure 4 depicts a dimension tree for glaucoma. We can see that the dimensions *IOP reduction* and *conjunctival hyperemia* have weights of 1 because they are leaf nodes and therefore there are no other dimensions with which they could be compared. Next, both *efficacy* and *safety* have weights of 0.5 meaning that both dimensions are equally important in this example.

**Fig. 5:**
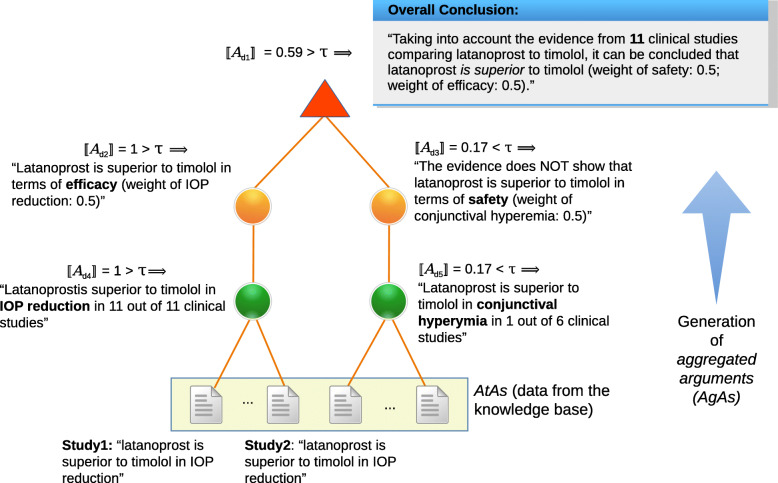
Example of the construction of a DIAeT for glaucoma. The confidence acceptance threshold used in this example is *τ*=0.5

Figure 5 depicts the construction of a DIAeT derived from the dimension tree in Fig. 4. The weight of all the atomic arguments is 1. The next level in the recursive process corresponds to the leaf nodes of the dimension tree (i.e., *d*_4_ and *d*_5_). For *IOP reduction* (*d*_4_), there are 11 out of the 11 clinical trials that state that latanoprost is more effective in reducing IOP than timolol, such that $\phantom {\dot {i}\!}\llbracket \mathcal {A}_{d_{4}} \rrbracket =1$ (i.e., (11/11)). For *conjunctival hyperemia* (*d*_5_), only in one of the six clinical trials that report this adverse effect, it was found that fewer patients suffered conjunctival hyperemia when applying latanoprost, such that $\phantom {\dot {i}\!}\llbracket \mathcal {A}_{d_{5}}\rrbracket = 0.17$ (i.e., (1/6)). Further, $\phantom {\dot {i}\!}\llbracket \mathcal {A}_{d_{2}} \rrbracket =1$ and $\phantom {\dot {i}\!}\llbracket \mathcal {A}_{d_{3}}\rrbracket =0.17$ because the weights of their children nodes (*d*_4_ and *d*_5_ respectively) are 1. Finally, $\phantom {\dot {i}\!}\llbracket \mathcal {A}_{d_{1}}\rrbracket =0.59$ is the result of the weighted sum of $\phantom {\dot {i}\!}\llbracket \mathcal {A}_{d_{2}} \rrbracket + \llbracket \mathcal {A}_{d_{3}}\rrbracket $ (i.e., (0.5∗1)+(0.5∗0.17)=0.59)^1^. We thus obtain the following conclusions:

- *Efficacy*: “the evidence shows that latanoprost is superior to timolol”, as $\phantom {\dot {i}\!}\llbracket \mathcal {A}_{d_{4}}\rrbracket =1 > 0.5 = \tau $.
- *Safety*: “the evidence does **not** show that latanoprost is superior to timolol”, as $\phantom {\dot {i}\!}\llbracket \mathcal {A}_{d_{3}}\rrbracket =0.17 < 0.5 = \tau $.
- *Overall conclusion*: “latanoprost is superior to timolol”, as $\phantom {\dot {i}\!}\llbracket \mathcal {A}_{d_{1}}\rrbracket =0.59 > 0.5 =\tau $

### Exploration of other scenarios (Sensitivity analysis)

Our approach allows to modify the weights of the dimensions and/or exclude certain evidence points. For example, studies that are biased or where the methodology applied is unclear, can be excluded by adjusting the parameters. One could also explore other scenarios (or “what-if” simulation) by filtering different criteria, such as publication year, duration of the study, and number or age of the participants in the clinical trials.

In the previous example, we could for instance explore other scenarios by assigning a higher weight of 0.7 to *safety* and a lower weight of 0.3 to *efficacy*. The new weights would generate **different degrees of confidence**. For example, the degree of confidence for $\phantom {\dot {i}\!}{A}_{d_{1}}$ would be now $\phantom {\dot {i}\!}\llbracket \mathcal {A}_{d_{1}}\rrbracket = 0.3 * 1 + 0.7 * 0.17 = 0.42$, and since 0.42<0.5, then the new overall conclusion would be opposite to the one obtained before:

*Overall conclusion*: “it can **not** be concluded that latanoprost is superior to timolol”, as $\phantom {\dot {i}\!}\llbracket \mathcal {A}_{d_{1}}\rrbracket =0.42 < 0.5 =\tau $

Further, if one excludes a study that compares the two drug treatments but that does not mention any result about conjunctival hyperemia (e.g., Mishima et al.,1996 in the tool demo), then the degree of confidence of $\phantom {\dot {i}\!}\mathcal {A}_{d_{4}}$ would change to 0.9 (i.e., 10/11). In contrast, $\phantom {\dot {i}\!}\llbracket \mathcal {A}_{d_{5}}\rrbracket $ would remain the same as 0.17 (i.e., 1/6). As a consequence: $\phantom {\dot {i}\!}\llbracket \mathcal {A}_{d_{2}}\rrbracket =0.9, \llbracket \mathcal {A}_{d_{3}}\rrbracket =0.17$, and $\phantom {\dot {i}\!}\llbracket \mathcal {A}_{d_{1}}\rrbracket =0.53$. Thus, the overall conclusion would change to “latanoprost is superior to timolol”. Table 3 summarizes the given example in which we can observe that when *safety* has a significantly higher weight than *efficacy* (e.g., 0.7 vs. 0.3), the overall conclusion changes to *“It* ***cannot*** *be concluded that latanoprost is superior to timolol”*. Otherwise, the conclusion indicates that *“Overall, the evidence showed that latanoprost is superior to timolol”*, including the case when one study is excluded.

**Table 3:** Conclusions generated with different settings

Weights	No. CTs	Conclusions
E50/S50	11/11	Overall: Lat > Tim ; Efficacy: Lat > Tim ; Safety: Lat ≯ Tim
E70/S30	11/11	Overall: Lat > Tim ; Efficacy: Lat > Tim ; Safety: Lat ≯ Tim
E30/S70	11/11	Overall: Lat ≯ Tim ; Efficacy: Lat > Tim ; Safety: Lat ≯ Tim
E50/S50	10/11	Overall: Lat > Tim ; Efficacy: Lat > Tim ; Safety: Lat ≯ Tim

E/S stands for efficacy/safety weights, Lat(anoprost), Tim(olol), No. CTs is the number of studies considered out of the total available studies, > means “ *treatment*_1_ is superior to *treatment*_2_”, and ≯ means “it can NOT be concluded that *treatment*_1_ is superior to *treatment*_2_”

#### Different acceptance thresholds

The user can also explore the conclusions generated according to different acceptance thresholds. For example, Table 4 shows the conclusions generated according to different threshold ranges. This example compares two kinds of insulin treatments for a T2DM case, where balanced dimension weights and no evidence filters are considered. It can be seen that the low thresholds lead to the conclusion stating that IGlar is superior to NPH insulin overall and with respect to safety and efficacy. Thresholds between 0.45 and 0.70 lead to the conclusion that the superiority of IGlar over NPH with regard to efficacy is not supported by the available evidence. Stricter thresholds ranging from 0.71 to 1 lead to the conclusion that the superiority of IGlar over NPH insulin overall and in terms of efficacy is not supported by the given evidence.

**Table 4:** Conclusions generated according to different acceptance threshold ranges

Threshold range	Overall Conclusion	Safety Conclusion	Efficacy Conclusion
[0,0.44]	IGlar > NPH insuline	IGlar > NPH insuline	IGlar > NPH insuline
[0.45,0.70]	IGlar > NPH insuline	IGlar > NPH insuline	IGlar ≯ NPH insuline
[0.71,1]	IGlar ≯ NPH insuline	IGlar > NPH insuline	IGlar ≯ NPH insuline

**Fig. 6:**
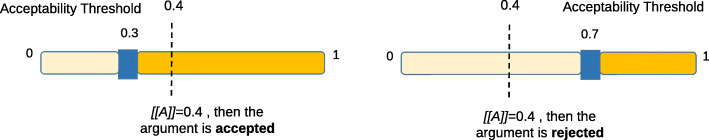
Example of confidence acceptance threshold. An argument is accepted if its degree of confidence *[[A]]* is not less than a given acceptance threshold, and rejected otherwise

Figure 6 depicts an example of the effect of changing the acceptance threshold. When the degree of confidence of an argument is not less than the acceptance threshold, then the argument is accepted, otherwise is rejected. The higher the threshold (i.e., closer to one), the stricter the acceptance of the argument becomes. In the opposite direction (i.e., closer to zero), the lower the threshold, the less restrictive the acceptance becomes.

**Fig. 7:**
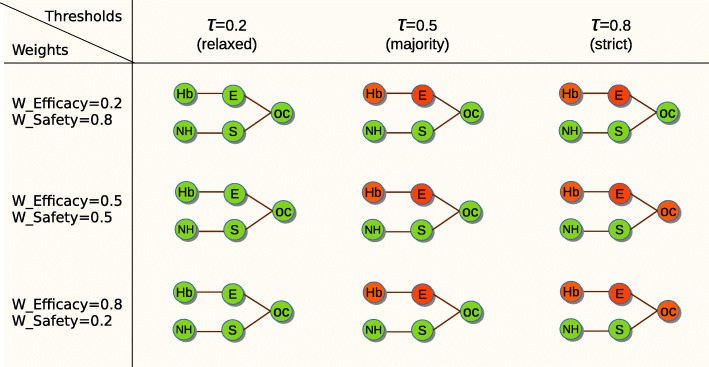
Trees generated with different confidence acceptance thresholds and weights. Where OS: Overall Conclusion, E: Efficacy, S: Safety, Hb: reduction of HbA1c, NH: Nocturnal hypoglycemia. The nodes in green are accepted arguments and in red rejected arguments on the superiority of IGlar insulin

Figure 7 shows the DIAeTs generated when using relaxed, majority and strict acceptance thresholds and three different dimension weight configurations to generate arguments on the superiority of the IGlar insulin treatment over the NPH insulin treatment. The threshold represents an acceptance condition for this statement, which implies the relative share of clinical evidence that supports (i.e., agrees with) the argument at the overall conclusion node, and the arguments at the dimension nodes that correspond to sub-conclusions. Setting the confidence threshold to 1 (strict) requires the evidence to be unanimous without any contradicting results. Setting the threshold to 0.5 (majority) requires the majority of studies to support the conclusion, while a value between 0 and 0.5 is very “lenient”, leading do the generation of arguments given very weak evidence. Along the table in Fig. 7, we can see that the stricter the threshold is, the more red nodes that are in the generated tree, that is, the more superiority arguments are rejected. Whereas with more relaxed thresholds, there are more green nodes, meaning that more superiority arguments are accepted.

### The DIAeT approach implemented as a web tool

**Fig. 8:**
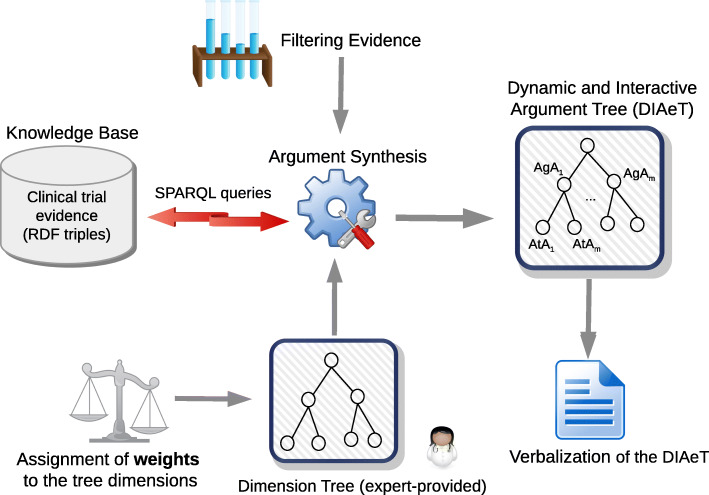
Overview of the DIAeT framework

The DIAeT approach has been implemented as a web tool as a proof of concept to support its evaluation with end users. Figure 8 provides an overview of the steps in the processing of the implemented method. The knowledge base that contains the clinical trial information and the weighted dimension tree are the starting-point for the system. The evidence is retrieved from the knowledge base via predefined SPARQL queries that are aligned with the dimensions in the dimension tree. Based on these elements, an argument synthesis process, in which evidence can also be filtered, generates a DIAeT that represents a nested conclusion about the superiority of some therapies compared to other therapies. The DIAeT is verbalized relying on domain-specific templates that make the conclusion accessible to the user. By defining filters or modifying weights, the users can interactively change the generated argument tree and thus explore the impact of certain choices on the synthesis of results.

**Fig. 9:**
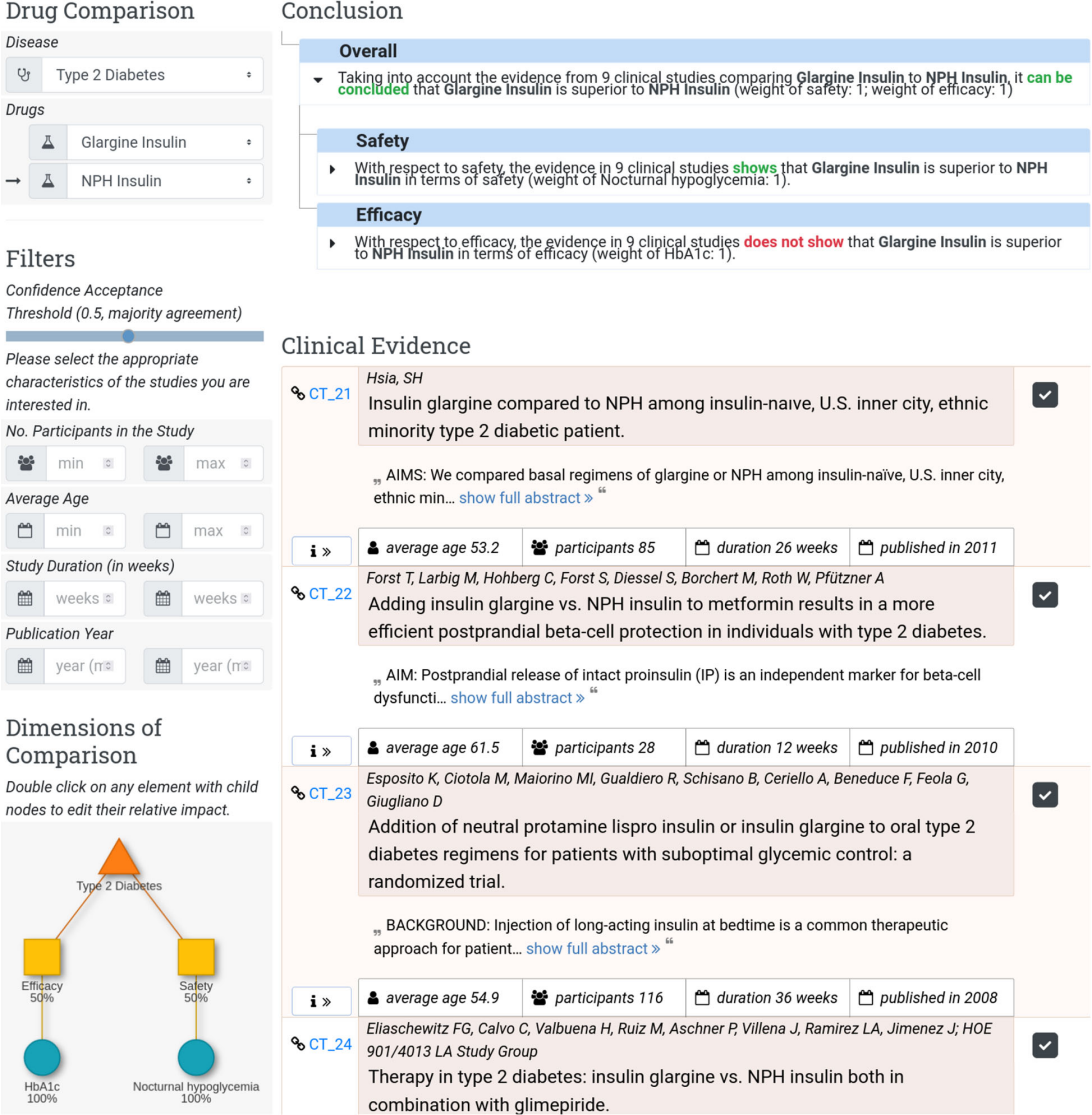
User interface of the DIAeT web tool. Left: evidence filters, confidence threshold (in the red square), and dimension tree weights. Right: generated conclusion tree and clinical evidence table

Figure 9 depicts the user interface of the DIAeT tool. The user can select treatments to compare, set the confidence acceptance threshold, and assign the weights for each dimension of a predefined dimension tree^2^. The reached conclusion for each dimension is represented in a hierarchical fashion along the hierarchically ordered criteria in the dimension tree. Each section can be expanded/hidden interactively. At the lowest level, the atomic arguments are displayed and it is indicated whether they support or contradict the conclusion. Supporting statements are displayed in green color and contradictory statements in orange.

**Fig. 10:**
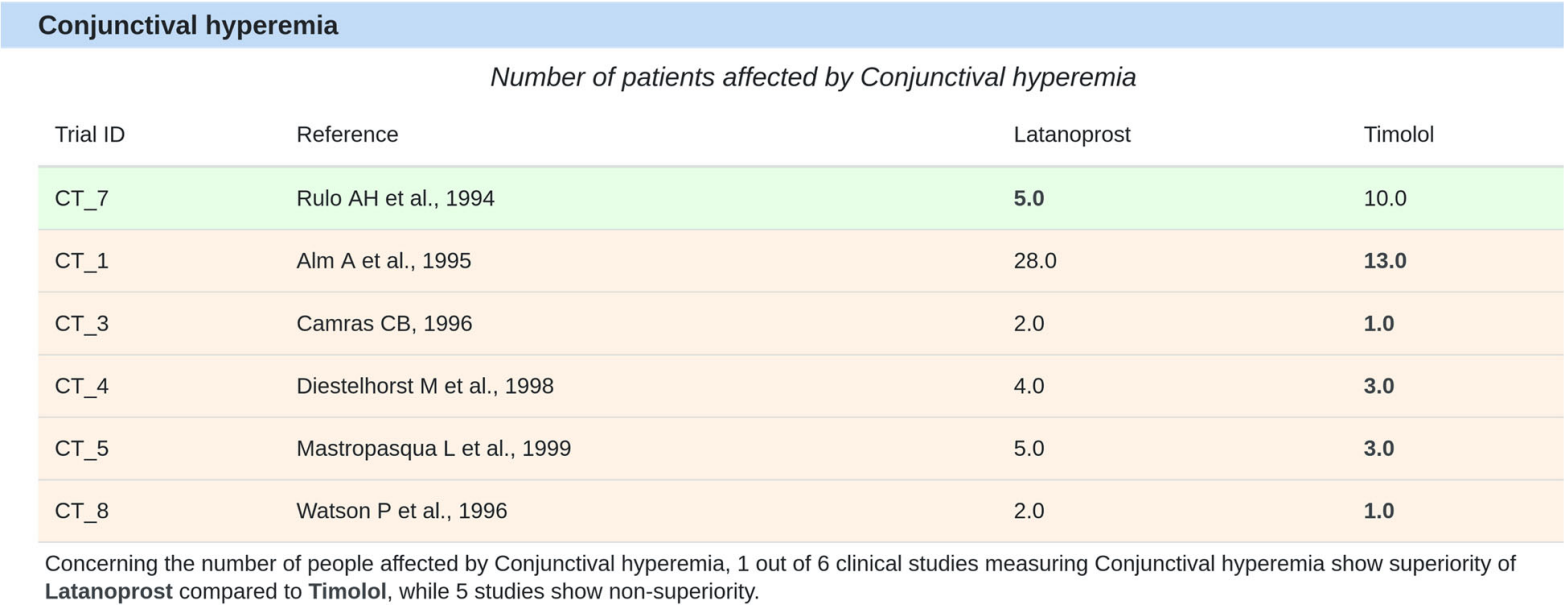
Atomic arguments for conjunctival hyperemia. For each atomic argument, contradictory (attacks) and supportive information is displayed. The values in bold font denote “superiority” of the respective drug (i.e., the drug that provokes less cases of conjunctival hyperemia). Supportive arguments are in green and contradictory arguments in orange

**Fig. 11:**
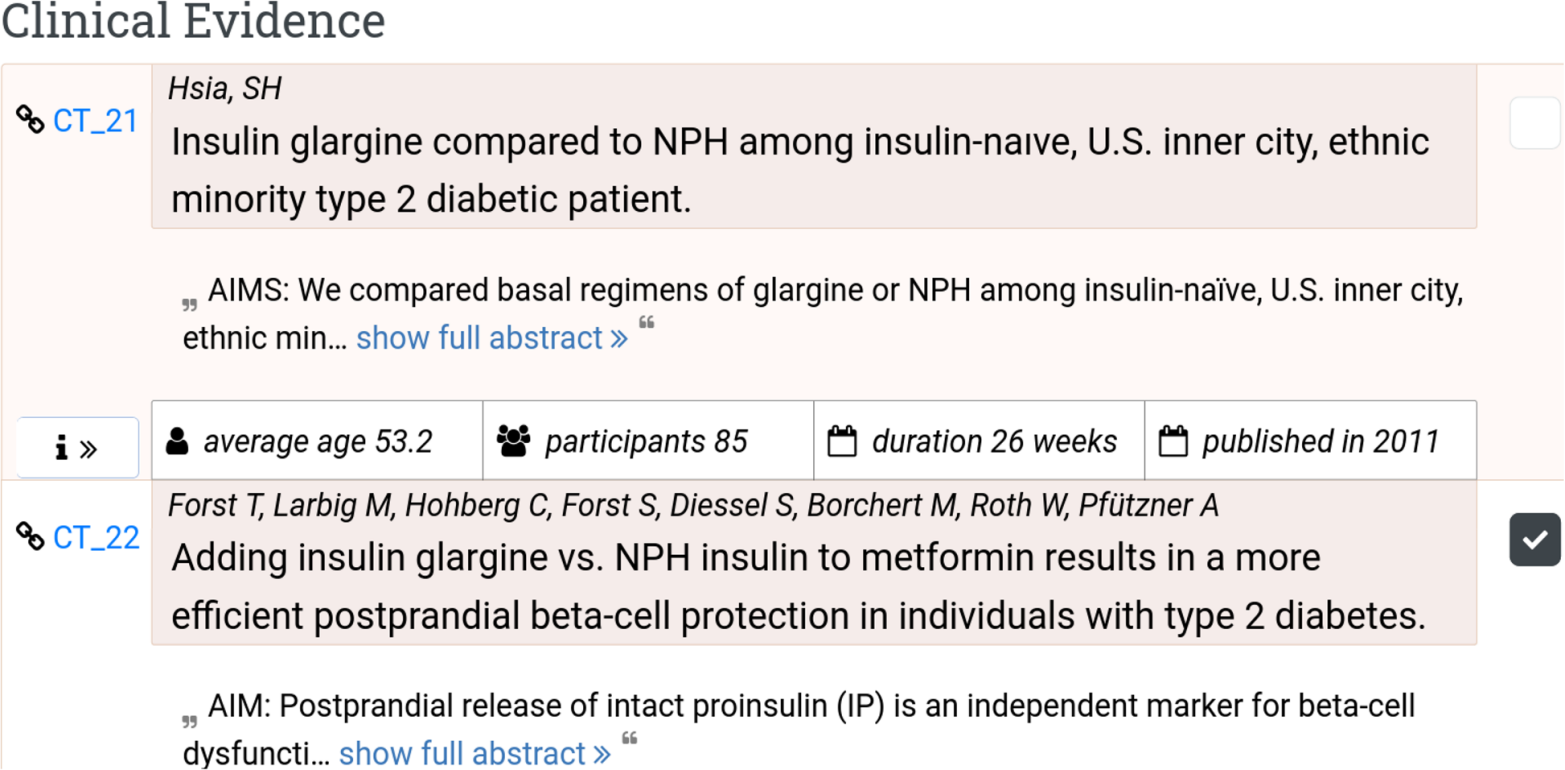
Clinical evidence table. The unticked study (on top) is not considered in the generation of the DIAeT

Figure 10 shows an example for *conjunctival hyperemia* where there are five atomic arguments attacking a single supportive evidence (study CT_7). Supportive arguments in this example are those that state that latanoprost causes less *conjunctival hyperemia* cases than timolol, while attacking arguments are those that imply a contradiction to the supportive arguments by stating that either latanoprost causes more *conjunctival hyperemia* cases, or that there are equal number of cases caused by both drugs (i.e., “latanoprost is not superior to timolol”). The evidence used to generate the DIAeT is displayed in the clinical evidence table. In this table, the user can find more information about the clinical trials, such as duration, number of patients, sources of possible biases, etc. (see Fig. 11)

Once the conclusions are generated, the tool allows the user to explore different scenarios by changing parameters (e.g. publication year, number and age of the participants, etc.), weights, confidence threshold, and exclude/include clinical studies (i.e., rebuttal of data), and then re-generate the conclusions. For example, specific studies can be excluded from the considered evidence if the user deems that the study does not meet certain criteria. All the studies that are considered by the system as supporting evidence are ticked in the evidence table. The user can then decide to untick them if he wants to explore what happens by not including them. Figure 11 depicts an example in which there are two studies in the evidence table. Only the ticked study will be considered in the construction of the arguments^3^.

Although the final decision on the best treatment is made by medical expert users, the method implemented as a tool would help them in the exploration of the information by, for example, narrowing the search space or helping her to understand under which conditions and assumptions it can be assumed that a certain treatment is superior to other treatments. If the medical experts find some interesting, unexpected or contradictory conclusions, they can directly check possible explanations for these conclusions in the published clinical trials.

## Results

In this section, we first describe the results of two use cases designed to evaluate whether our method is able to produce similar conclusions to the ones of published systematic reviews. Next, we describe a survey conducted to assess the possible benefits and the use of our method as a web tool.

### Use cases

As a proof of concept of our method, we present two use cases. One use case is on glaucoma and another on Type 2 Diabetes Mellitus (T2DM). The aim is to analyse whether our method is able to generate similar conclusions compared to the ones reached in the existing published systematic reviews selected for these two diseases.

We first formalized the evidence of each of the trials considered in the respective systematic reviews for glaucoma and T2DM [19, 20] as described in the section “The C-TrO ontology and knowledge base”.

We then defined the dimension trees for each disease. Both dimension trees contain dimensions for *efficacy* and *safety*, which are common aspects of interest in clinical trials. The sub-dimensions of these dimensions were specified according to the main endpoints (i.e., outcomes and adverse effects) analyzed for each disease when applying the medical treatments studied in the respective systematic reviews. Although our approach allows any number of dimensions, we only include one endpoint and one adverse effect as sub-dimensions for simplifying the use cases. Equal weights were assigned to *efficacy* and *safety* (i.e., 50% each). Table 5 summarizes the characteristics of the use cases.

**Table 5:** Characteristics of the use cases

Disease	SRs	No. RCTs	Dimensions {sub-dimensions}	Compared drugs
Glaucoma	Zhang et al. [19]	11	efficacy {IOP reduction}, safety {conjunctival hyperemia}	latanoprost / timolol
T2DM	Rys et al. [20]	9	efficacy {reduction of HbA1c}, safety {hypoglycemia}	insulin glargine / NPH insulin

The inferential criteria for *efficacy* and *safety* used in both use cases to establish superiority of a treatment over other treatments are the following (Cf. [21]): Efficacy: If the *drug*1 treatment changes a given disease indicator in the desired direction from the baseline – in terms of an aggregation method – in greater magnitude than the *drug*2 treatment, then the *drug*1 treatment is more effective than the *drug*2 treatment. Safety: If the administration of the *drug*1 treatment leads to fewer incidences of a given adverse effect compared to the administration of the *drug*2 treatment, then the *drug*1 treatment is safer than the *drug*2 treatment with respect to the given adverse effect.

Note that these criteria can be changed or augmented to include more complex cases. For example, the efficacy criterion could include combined therapies that involve the application of more than one drug treatment to the same interventional arm at different time points and duration. However, because the use cases presented here refer to single-drug treatments, we use a simple efficacy criterion that involves a single treatment.

Given the initial configuration, the respective DIAeTs were generated without applying any filter to the evidence retrieved from the knowledge base.

For **glaucoma**, the conclusions obtained with the DIAeT tool for efficacy, safety, and overall superiority when comparing latanoprost to timolol are:

- “The evidence in 11 clinical studies shows that latanoprost *is superior* to timolol in terms of **efficacy** (weight of diurnal IOP: 1).”
- “The evidence in 11 clinical studies *does not show* that latanoprost is superior to timolol in terms of **safety** (weight of conjunctival hyperemia: 1).”
- “Taking into account the evidence from 11 clinical studies comparing latanoprost to timolol, it can be concluded that latanoprost *is superior* to timolol (weight of safety: 1; weight of efficacy: 1)”.

The conclusions reached in the systematic review by Zhang et al. [19] with respect to efficacy in terms of IOP reduction and safety in terms of conjunctival hyperaemia, and overall are respectively:

- “Latanoprost showed better IOP lowering effects than timolol with an additional 4–7% reduction. The differences were all statistically significant except for the result from a single 12 months study.”
- “Latanoprost caused hyperaemia and iris pigmentation in more patients than timolol. The risk for hyperaemia was over twice that seen with timolol (RR = 2.20, 95% CI 1.33,3.65).
- “Latanoprost *is superior* to timolol for reducing intraocular pressure.”

Zhang et al. conclude that, **overall**, latanoprost is superior to timolol despite the different side effects that it might provoke. This conclusion is in line with the ones obtained by our tool that states that **in general** latanoprost is superior to timolol, and in particular in terms of efficacy considering IOP reduction. However, in terms of safety, considering hyperaemia, latanoprost is not superior to timolol.

For **T2DM**, the conclusions on efficacy, safety and overall superiority generated by our tool when comparing Glargine Insulin (IGlar) to NPH Insulin are:

- “The evidence in 9 clinical studies *does not show* that Glargine Insulin is superior to NPH Insulin in terms of **efficacy** (weight of HbA1c: 1).”
- “The evidence in 9 clinical studies shows that Glargine Insulin *is superior* to NPH Insulin in terms of **safety** (weight of nocturnal hypoglycemia: 1).”
- “Taking into account the evidence from 9 clinical studies comparing Glargine Insulin to NPH Insulin, it can be concluded that Glargine Insulin *is superior* to NPH Insulin (weight of safety: 1; weight of efficacy: 1).”

Therefore, the evidence shows that overall IGlar *is superior* to NPH insulin despite IGlar *not being superior* to NPH Insulin in terms of efficacy.

In the systematic review conducted by Rys et al. [20], the conclusions with respect to efficacy in terms of reduction of HbA1c levels, safety in terms of nocturnal hypoglycemia, and overall are respectively:

- “The study demonstrated a difference in HbA1c reduction in favor of twice daily NPH insulin...”
- “The analysis of individual endpoints demonstrated comparable reduction of HbA1c in each arm, but with concomitantly lower rate of symptomatic and nocturnal hypoglycemia in IGlar group.”
- “In conclusion, for the majority of examined efficacy and safety outcomes, IGlar use in T2DM patients was superior or at least non-inferior to the alternative insulin treatment options.” (i.e., NPH insulin and the other insulins studied).

These conclusions coincide with the ones generated by our tool in that overall IGlar is superior to NPH insulin, in particular with respect to safety and in reducing the risk of nocturnal hypoglycemia, but not superior in efficacy in terms of the reduction of HbA1c levels.

The presented use cases include only some relevant dimensions for each disease. However, other important dimensions could be defined. For example, for T2DM the dimension long-term harm can be added, which may include conditions such as myocardial infarction, stroke, or kidney failure. Although these use cases contain few dimensions (i.e., study endpoints), they demonstrate that the method can automatically generate similar conclusions for these endpoints as the conclusions reached in manually produced systematic reviews. Moreover, a main benefit of our approach is that it supports exploring the consequences of different preferences and weights interactively and reasoning under different assumptions.

### Evaluation of the web application

To evaluate the use and acceptance of the DIAeT tool, we conducted an on-line survey in which 17 medical experts (13 men and 4 women) from different hospitals in Germany took part. The participants were between 25 and 54 years old, most of them in the range of 35-44 years. They had different medical specializations and at least one year of experience in their fields (number of participants per specialization: anesthesiology (4), pediatrics (2), general medicine (2), emergency medicine (2), cardiology (2), oral surgery (1), otorhinolaryngology (2), and neurology (1), internal medicine (1)).

The survey consists of three sections, and most of the responses are based on a 5-points Likert-scale [22] where 1: strongly disagree, 2: disagree, 3: neither agree nor disagree, 4: agree, 5: strongly agree. The first section is concerned with testing the level of understanding of the central aspects of the method regarding the conclusion of the system, how the filters and weight modifications would affect the conclusion, etc. The second section is about the benefit of using the tool in terms of exploring and summarizing clinical evidence. The last section is for assessing the usability of the method as a web tool.

The results for the first section are shown in Table 6. Most of the answers agree or strongly agree with the statements relative to the aim of the tool and how to use it, including the objective and setting-up of the filters on the clinical evidence to be considered, how to change the dimension weights, and how these weights influence the resulting conclusions. The responses of the participants suggest that they could understand how the conclusions are generated based on the included/excluded studies. However, the low percentage of strong agreement (17.67%) on the sufficiency of the metadata about studies (question 10) suggests that the participants would need more information to be able to judge whether the inclusion of the study is warranted. In general, these results suggest that the goal and use of the tool were clear for the users.

**Table 6:** Section 1 of the survey: questions related to the understanding of the objective and use of the tool, and the percentages obtained for each type of answer

Questions	%S-1	%S-2	%S-3	%S-4	%S-5
1. The motivation and goals behind the development of the tool are clear to me.	0.00	5.88	5.88	23.53	64.71
2. The explanations in the video on using the tool are understandable.	0.00	5.88	11.76	11.76	70.59
3. I understand how to set a filter on the clinical studies being considered.	5.88	0.00	5.88	17.65	70.59
4. I understand what setting a filter does.	5.88	0.00	0.00	23.53	70.59
5. I understand how to change the weights of the individual dimensions.	11.76	5.88	5.88	23.53	52.94
6. I understand the influence of the weighting of the different dimensions (safety, efficacy) on the conclusion of the system.	0.00	11.76	11.76	11.76	64.71
7. The conclusion of he system is clear and understandable.	0.00	0.00	17.75	29.41	52.94
8. It is understandable how the system comes to the conclusions based on the selected clinical studies.	0.00	11.76	23.53	11.76	52.94
9. It is clear how to include or exclude a study in the calculation of the conclusion.	0.00	0.00	0.00	41.18	58.82
10. The metadata shown for the individual studies is sufficient to assess the relevance of the study with regard to its inclusion.	0.00	11.76	35.29	35.29	17.65

S-1: strongly disagree, S-2: disagree, S-3: neither agree nor disagree, S-4: agree, S-5: strongly agree

Table 7 shows the answers for the questions in the second section. Most of the participants agree or strongly agree with the given statements. This suggests that the participants found the tool useful for the exploration of clinical evidence. The perceived benefits of the tool include the time-efficient comparison of drug treatments, decision-making-support in cases where insufficient or outdated information is provided in clinical guidelines, or where no guidelines exist at all (64.71% strongly agree). However, the participants were rather unsure to agree on whether the tool would help when the characteristics of a particular patient deviate from the average population studied in the guidelines (question 7). This suggests that further information (e.g., study protocol and population characteristics) is needed to decide on the best treatment for an individual patient.

**Table 7:** Section 2 of the survey: questions related to the benefits of the tool and the percentages obtained for each type of answer

Questions	%S-1	%S-2	%S-3	%S-4	%S-5
*Imagine that the system and all the relevant studies were available for your subject:*					
1. I can imagine using this system in my daily work to support therapy decisions.	0.00	17.65	17.65	35.29	29.41
2. The system would help me to determine the best therapy option based on the current studies.	0.00	11.76	23.53	41.18	23.53
3. I believe that this system can save me time if I have to choose between two treatments based on the current study situation.	0.00	0.00	17.65	41.18	41.18
*I can imagine a good use of the system in the following situations:*					
4. When there are no guidelines.	0.00	0.00	5.88	29.41	64.71
5. As a complement to the existing guidelines when the information in the guideline is insufficient.	0.00	0.00	23.53	35.29	41.18
6. When guidelines are outdated.	0.00	0.00	23.53	41.18	35.29
7. When the characteristics of a given patient deviate significantly from the average population in the guidelines.	0.00	11.76	35.29	29.41	23.53

S-1: strongly disagree, S-2: disagree, S-3: neither agree nor disagree, S-4: agree, S-5: strongly agree

In the third section, we use the System Usability Scale (SUS) [23], which is a standard method to measure system usability. The SUS consists of the ten questions presented in Table 8 that are answered on a 5-points scale. For details on how the SUS score is calculated, the reader is referred to Brooke et al. [23].

**Table 8:** System Usability Scale (SUS) questions

N.	Questions
1	I think that I would like to use this system frequently
2	I found the system unnecessarily complex
3	I thought the system was easy to use
4	I think that I would need the support of a technical person to be able to use this system
5	I found the various functions in this system were well integrated
6	I thought there was too much inconsistency in this system
7	I would imagine that most people would learn to use this system very quickly
8	I found the system very cumbersome to use
9	I felt very confident using the system
10	I needed to learn a lot of things before I could get going with this system

The responses and obtained scores are presented in Table 9. The average SUS score is 76.91 (95%CI, [69.91, 83.91]), which indicates that the participants found the web tool easy to understand and operate.

**Table 9:** Responses to the System Usability Scale (SUS) questions of the 17 participants. The calculated SUS scores are in the last column

Participants	Q1	Q2	Q3	Q4	Q5	Q6	Q7	Q8	Q9	Q10	SUS
p1	3	1	5	1	4	1	5	1	5	1	92.50
p2	4	1	5	2	5	1	5	1	4	1	92.50
p3	4	1	5	1	5	2	5	1	5	1	95.00
p4	4	3	3	1	3	3	5	2	4	1	72.50
p5	3	1	4	1	5	2	5	1	3	1	85.00
p6	4	1	5	1	4	2	5	2	4	1	87.50
p7	3	3	3	2	3	4	4	3	3	4	50.00
p8	5	1	5	1	4	1	5	1	4	1	95.00
p9	3	2	3	2	3	2	3	2	2	3	57.50
p10	3	2	3	2	4	3	3	3	4	2	62.50
p11	3	2	4	2	4	2	4	2	3	2	70.00
p12	3	1	5	1	4	2	5	1	5	1	90.00
p13	2	3	4	1	4	3	4	2	4	2	67.50
p14	4	2	4	1	4	2	4	1	3	3	75.00
p15	3	1	5	1	2	2	5	1	5	1	85.00
p16	3	2	3	1	2	3	4	2	4	2	65.00
p17	4	2	4	3	4	3	4	2	3	3	65.00
Average SUS											76.91

Overall, the results of the conducted survey suggest that our method implemented as a web tool can be useful for medical practitioners to support the exploration and summarization of clinical evidence.

## Discussion

The creation of systematic reviews is a long-term process requiring substantial personnel and efforts [24], and keeping them up-to-date represents a significant challenge [25, 26]. To alleviate this situation, the International Collaboration for the Automation of Systematic Reviews (ICASR) is exploring tools and methodologies that can partially automatize or at least reduce the effort involved in the creation of systematic reviews [27]. The DIAeT method can support different steps in the creation of systematic reviews, such as supporting the synthesis process by helping users to explore and analyze different settings (populations, trial designs, etc.) and to determine which trials contain evidence worthy to be included in the review and useful in the analysis and generation of conclusions about the superiority of treatments. Furthermore, the DIAeT tool can be used to support the formulation of review questions since the users can try different configurations that help them to identify questions of interest. Prioritizing questions can save time and avoid duplicated and irrelevant questions. In the screening of abstracts and titles of published trials, the DIAeT tool could help to quickly exclude several studies that may be irrelevant for the systematic review. The DIAeT tool could also help to close the evidence-practice gap (or *knowing* to *doing* gap) by the aggregation of contradictory or incomplete clinical evidence - according to the criteria and preferences of the expert users - that leads to the generation of textual and justified conclusions. Further, it could help to facilitate the selection of evidence and the calibration of parameters that allows a more effective production of systematic reviews.

The DIAeT approach presented in this article is argument-based and similar to the practical aspect of Toulmin’s model of argumentation [7]. Toulmin’s model has gained relevance in Evidence-Based-Medicine because it is able to bring explicitness to the role of evidence in clinical reasoning [28]. It is a practical approach to argument analysis that identifies interrelated components of an argument in a given order and structure. The model focuses on the justificatory aspect of argumentation by effectively representing justifications (or warrants) that support a given conclusion. It also makes the relationship between the claims, their evidential support, and the possibly conflicting information explicit. The DIAeT approach to synthesizing the results of clinical trials is similar to Toulmin’s model in that it focuses on the justification of the conclusions warranted by the available evidence.

The abstract framework of Hunter and Williams [6] is also concerned with the aggregation of clinical evidence. It presupposes a certain aggregation level of such evidence in the sense that the relative risk values, used as evidence, have already been calculated based on multiple studies. In contrast, our DIAeT method is applicable to the raw evidence available in clinical studies. Besides, the DIAeT method explicitly deals with confidence degrees that allows to express uncertainty that is key when evidence not always consistently supports a conclusion. In contrast, Hunter and Williams’ framework does not allow to represent the level of inconsistency and uncertainty in the evidence. Furthermore, the actual reasons for treatment superiority can not be read off from the final argument graph in the framework of Hunter and Williams, while the full nested argument can be inspected by the users in our tool, giving the full reasons for the overall conclusion on treatment superiority.

Regarding the semantic technology aspect, the DIAeT method requires clinical trial information formalized in a knowledge base following a suitable ontology and the integration of semantic vocabularies. While this may seem to be a bottleneck for the large-scale implementation of the proposed approach, there are currently signs that this will indeed not be a limiting factor. For one, it is possible to develop intuitive interfaces that guide authors of clinical trial publications, voluntaries or crowd-sourcing participants to describe the main results of a clinical trial with respect to a given ontology. In a recent study [29], we have shown that the semantic modelling of clinical trials based on the C-TrO ontology is feasible using an editor called CTrO-Editor that has been designed for this purpose. We showed that medical students take a couple of hours to capture the information of a clinical study using CTrO-Editor. Furthermore, other clinical trial evidence sources could be linked or integrated to the clinical trial publication knowledge base, such as the information from clinicaltrials.gov.

The method is generic since the core elements to build the argument tree, such as dimension tree and superiority criteria, can be adapted to other health conditions or diseases. For this purpose, it would be necessary that the knowledge base contains the information relative to these conditions. If the method was used in a context other than clinical trials, a knowledge base with the appropriate information would be needed.

Although recent text mining and NLP solutions have progressed in the extraction of the core ‘evidence tables’ from clinical trial publications [30], a limitation of these methods is that they need training data for different therapy areas/diseases that a system has to support. Further, these systems would require handling the errors introduced by the information extraction systems, or at least a process by which errors can be corrected by the research community. This would also require appropriate interfaces, as mentioned before.

## Conclusions

In this article, we have presented a method that facilitates the synthesis of clinical evidence via an argument-based approach that automatically generates a tree-shaped conclusion on the basis of clinical trials semantically captured in a knowledge base. Our approach allows users to explore the impact of filtering the evidence as well as of setting weights for different comparison dimensions interactively and dynamically on the generated conclusion, thus supporting to carry out sensitivity analyses.

The method has been implemented as a web tool that can be adapted to different indications or therapeutic areas. The web tool allows users to systematically explore the implications of excluding certain points of evidence, or indicating relative preferences of endpoints via weight setting. Our argument-based approach has been shown to be able to generate conclusions that are comparable to those of the manually produced systematic reviews. It has also shown to be generic in the sense that it can be applied to different health conditions, as shown in the use cases presented in two different diseases.

Our evaluation with medical experts has revealed that the tool is easy to understand and use and that it has the potential to support experts in assessing the current evidence as a complement or extension to existing guidelines, helping them to reach better decisions. The method can also support the automation of systematic reviews, as explored by the International Collaboration for the Automation of Systematic Reviews (ICASR).

In future work, we intend to develop the methodology further to support the development of continually updated (“living”) systematic reviews. We will also develop information extraction methods that can automatically extract relevant evidence from published trials.

## Data Availability

The source code of the implementation of our method and the knowledge base are publicly available at: 10.5281/zenodo.5604516. The demo of the web-based tool is available at: https://scdemo.techfak.uni-bielefeld.de/ratio-argviz/.

## Abbreviations

ADDIS: Aggregation Data Drug System
AgA: Aggregated Argument
AtA: Atomic Argument
C-TrO: a Clinical Trial Ontology for aggregation
DIAeT: Dynamic Interactive Argument Trees
EBM: Evidence-Based Medicine
ICASR: International Collaboration for the Automation of Systematic Reviews
HbA1c: Glycated Haemoglobin
ICD: International Classification of Diseases
IGlar: Insulin Glargine
KB: knowledge base
MeSH: Medical Subject Headings
NLP: Natural Language Processing
NPH: Neutral Protamine Hagedorn (type of insulin)
PICO: Problem/Population, Intervention, Comparison, Outcome
PSINK: Preclinical Spinal Cord Injury Knowledge Base
RCT: Randomized Clinical Trial
RDF: Resource Description Framework
SNOMED CT: Systemized Nomenclature of Medicine – Clinical Terms
SCO: Study Cohort Ontology
SPARQL: standard RDF query language
SR: Systematic Review
SUS: System Usability Scale
T2DM: Type Two Diabetes Mellitus
XML: Extensible Markup Language

## Footnotes

1 Note that the normalization factor in our examples is 1 since the sum of the weights of the sub-dimensions of the given dimensions is always 1 for these examples.

2 The weights of sibling dimensions have to sum up 100%

3 A demo of our tool can be accessed as indicated in the “Availability of data and materials” section.
